# Aqueous-Methanol Extracts of Orange-Fleshed Sweet Potato (*Ipomoea* *batatas*) Ameliorate Oxidative Stress and Modulate Type 2 Diabetes Associated Genes in Insulin Resistant C2C12 Cells

**DOI:** 10.3390/molecules23082058

**Published:** 2018-08-17

**Authors:** Taiwo Betty Ayeleso, Khosi Ramachela, Emmanuel Mukwevho

**Affiliations:** 1 Department of Biochemistry, North West University, Private Bag X2046, Mmabatho 2735, South Africa; taiwo.ayeleso@gmail.com; 2 Department of Crop Science, North-West University, Private Bag X2046, Mmabatho 2735, South Africa; khosi.ramachela@nwu.ac.za

**Keywords:** oxidative stress, insulin resistance, palmitate, glutathione, diabetes, malonaldehyde

## Abstract

Edible plants such as sweet potato are sources of natural antioxidants that can be exploited in the management and treatment of insulin resistance. This present study investigated the effects of the extracts of an orange-fleshed sweet potato on oxidative stress biomarkers (glutathione status and lipid peroxidation) and activities of antioxidant enzymes (catalase, CAT and glutathione peroxidase, GPx) in palmitate-induced insulin resistant C2C12 cells. The intracellular antioxidant status of the cells was also measured using Ferric reducing antioxidant power (FRAP) and Trolox equivalent antioxidant capacity (TEAC) assays. Furthermore, this study determined the effect of the extracts on the regulation of some type 2 diabetes associated genes; glucose transporter 4 (*glut4*), Nuclear respiratory factor 1 (*nrf1*), Myocyte enhanced factor 2A (*mef2a*), Carnitine palmitoyltransferase 1 (*cpt1*) and Acetyl-CoA carboxylase 2 (*acc2*). The results showed a significant (*p* < 0.05) increase in intracellular GSH level, a significant reduction in the level of malonaldehyde and a significant improvement in the intracellular antioxidant status upon treatment of the insulin resistant cells with the extracts. The extracts were also able to positively modulate the expression levels of the type 2 diabetes associated genes. On the other hand, HPLC-MS analysis of the extracts showed the presence of polyphenols which could have contributed to the bioactivity of the extracts through their antioxidant effects.

## 1. Introduction

Insulin resistance is generally an impaired ability of insulin to stimulate glucose uptake and utilization [1]. It is the most powerful indicator of an imminent development of type 2 diabetes and therefore an important therapeutic target in the management of the disease [2]. Oxidative stress which occurs as a result of an imbalance between the productions of reactive oxygen species (ROS) and antioxidant defenses is a major pathological cause of several chronic diseases including diabetes [3]. Oxidative stress has been implicated in the development of insulin resistance and its progression into type 2 diabetes and its complications [4,5]. Therefore, the inhibition of oxidative stress is crucial in the prevention and management of insulin resistance and diabetes.

Another important factor involved in the development of insulin resistance is the reduced ability of skeletal muscle to adjust easily between glucose and fatty acid oxidation in response to homeostatic signals [6]. In the skeletal muscle cells, insulin-stimulated transport of glucose relies on a number of signaling components which results in the translocation of the glucose transporter 4 (GLUT4) to the cell surface [7]. GLUT4 is a transmembrane protein that allows the transport of peripheral blood glucose across the plasma membrane into the cell through facilitated diffusion [8]. The *glut4* gene promoter has a binding region for myocyte-enhanced factor 2A (*mef2a*) transcription factor to facilitate *glut4* expression [9], therefore, *mef2a* also functions as a major transcriptional regulator of glucose uptake metabolism. Another transcription factor that plays a role in glucose transport is the Nuclear respiratory factor 1 (*nrf1*) gene which is involved in the transcriptional activation of *mef2a* through peroxisome proliferator-activated receptor gamma coactivator 1 alpha (PGC-1α) [10,11]. *Glut4* and its transcription factors are important pharmacological targets in the management of glucose homeostasis.

Other than glucose, fatty acids, which are essential components of membrane lipids and a major source of energy, also play an important role in the pathogenesis of metabolic diseases, including type 2 diabetes [12]. Acetyl-CoA carboxylases (ACC) are important regulatory enzymes involved in the regulation of fatty acid synthesis and oxidation in response to dietary changes [13]. The two isoforms of ACC; *acc1* and *acc2* provide the substrate (malonyl CoA) for the synthesis and oxidation of fatty acid respectively; hence they both play important roles in fatty acid metabolism. Carnitine palmitoyl transferase I (*cpt1*), a mitochondrial transmembrane enzyme, also plays a significant role in the facilitation of long-chain fatty acid entry into the mitochondria for beta oxidation [14,15]. The modulation of the genes and transcription factors involved in glucose and fatty acid metabolism is an important measure in the management of type 2 diabetes.

Edible plants are a source of dietary components that can act as reducing agents against ROS [16] and prevent oxidative stress mediated diseases such as type 2 diabetes [17]. Sweet potato is a food crop that contains carbohydrates, proteins, carotenoids and polyphenols in different proportions depending on the cultivar or variety [18]. Carotenoids and anthocyanins are some of the bioactive compounds which are particularly abundant in the orange and purple fleshed varieties respectively [19,20]. Sweet potato has been found to be rich in polyphenolic compounds such as caffeic acid, quercetin and their derivatives [18]. Polyphenols are a group of phytochemicals which are considered highly potent antioxidant and bioactive compounds [21,22]. There are several literature evidence of the biological activities of sweet potato including its antioxidant and antidiabetic effects particularly the white fleshed varieties [23,24]. The in vitro antioxidant activities of sweet potato extracts have been demonstrated using different methods such as ferric reducing antioxidant power (FRAP), 2,2-diphenyl-1-picrylhydrazyl (DPPH) and Trolox equivalent antioxidant capacity (TEAC) assays [25,26] but the anti-oxidative activity of the extracts in an in vitro model of disease has not been explored. Nevertheless, in vivo antidiabetic studies have shown the ability of the extracts of white varieties of sweet potato to lower blood glucose level and improve insulin sensitivity in animal models [23,24] but there are little or no such report in the orange fleshed varieties. Furthermore, studies such as that of Alam and his colleagues [20] have investigated the chemical composition of orange fleshed sweet potato, and found that apart from the proximate composition and the pigment compound, carotenoids, they are also good source of polyphenols. However, most of the available data on the polyphenol contents of orange fleshed sweet potato are measured as the total polyphenol content [27,28]. Therefore, this study quantified specific bioactive phenolic compounds in the aqueous and aqueous-methanol extracts of orange-fleshed sweet potato. It went further to assess the effects of the aqueous-methanol extracts on intracellular oxidative stress and the expression level of genes associated with type 2 diabetes in insulin-resistant C2C12 skeletal muscle cells.

## 2. Results and Discussion

### 2.1. Quantification of Specific Polyphenols in the Extracts

**Figure 1:**
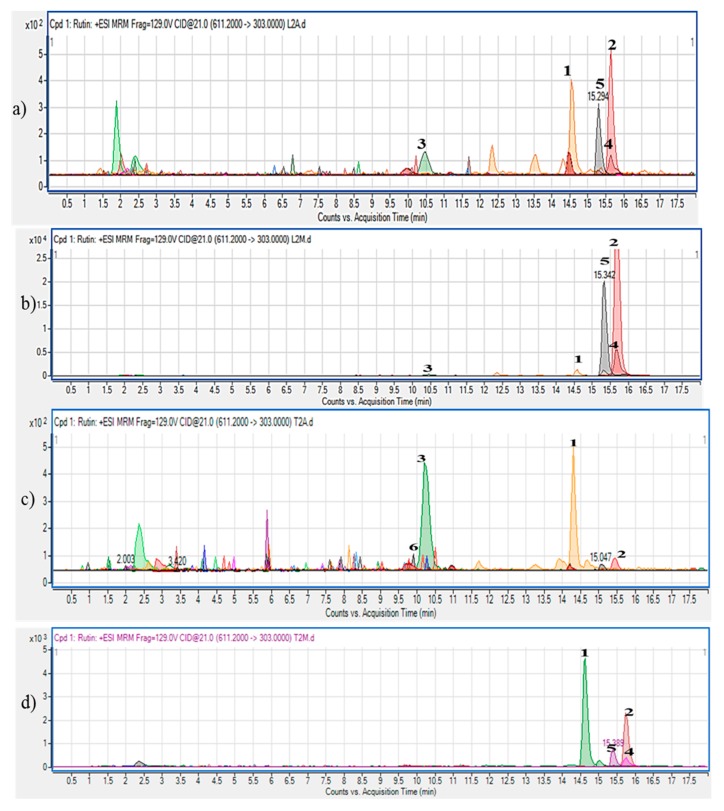
HPLC chromatograms of (**a**) aqueous extract of the leaves (**b**) aqueous-methanol extract of the leaves (**c**) Aqueous extract of the tubers (**d**) aqueous-methanol extracts of the tubers of orange fleshed sweet potato. 1—Caffeic acid, 2—Hyperoside, 3—Protocatechuic acid, 4—Quercetin, 5—Rutin, 6—Vanillic acid.

Characterization of both the aqueous and aqueous-methanol extracts of the orange fleshed sweet potato was carried out through HPLC-MS analysis to determine the level of some beneficial polyphenols in the extracts. The detected peaks in the chromatograms (Figure 1) were identified and quantified by comparing the retention time and peak area to that of known standards. The polyphenolic compounds were detected at varying concentrations in the extracts (Table 1). Generally, the most abundant phenolic compound in the extracts was hyperoside with the highest amount found in the aqueous-methanol extract of the leaves (139.83 mg/g extract). Hyperoside is a flavonoid compound which is commonly found in medicinal plants. Most Chinese herbal plants are rich in hyperoside which has been found to contribute significantly to the medicinal actions of these plants [29]. The presence of hyperoside has been reported in the aqueous-acetone extract of sweet potato leaves [30]. This study demonstrated the presence of hyperoside in the aqueous and aqueous-methanol extracts of both the leaves and tubers of the orange fleshed sweet potato, although, the amount is about 30-fold lower in the tubers. Caffeic acid is the most abundant compound in the tubers, the highest amount with a value of 19.40 mg/gextract was also found in the aqueous-methanol extract. Several studies have reported the presence of caffeic acid in the leaves and tubers of various cultivars of sweet potato [31,32]. Caffeic acid has been identified as a potent antioxidant agent in plants [33,34]. Olivier and colleagues [35] reported the presence of caffeic acid in species of *Arctopus* and *Alepidea* which are important South African medicinal plants. Rutin and quercetin were also found in considerable amounts in the aqueous-methanol extracts of the leaves (69.67, 23.36 mg/g extract) as well as in the tubers (2.91 and 1.76 mg/g extract) extracts respectively. However, the amount of rutin and quercetin is negligible in the aqueous extracts of both the tubers and leaves. The therapeutic effects of rutin and quercetin in type 2 diabetic conditions have been documented in different studies [36,37]. Generally, trace amounts of vanillic acid, isovanillic acid and protocatechuic acid were found in all the extracts. The importance of the influence of extraction solvents on the level of polyphenols found in plants was also demonstrated in this study. This is because, all the quantified compounds were in higher concentrations in the aqueous-methanol extracts of the samples than in the aqueous extracts with the exceptions of isovanillic and protocatechuic acid in the tubers. Thus, aqueous-methanol extracts of both the leaves and tubers were further used in the study to treat insulin resistant C2C12 cells.

### 2.2. Effect of Sweet Potato Extracts on Oxidative Stress in Insulin Resistant C2C12 Cells

#### 2.2.1. Effect of Sweet Potato Extracts on Total Glutathione Status and Lipid Peroxidation

**Figure 2:**
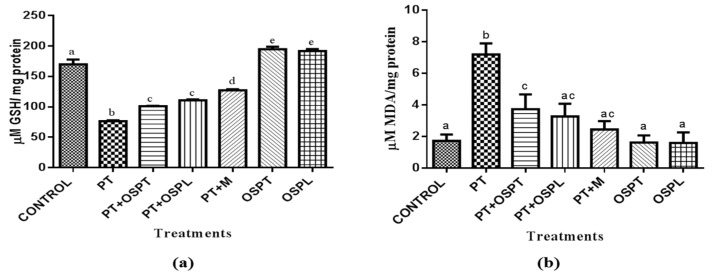
The effect of orange fleshed sweet potato extracts on (**a**) total glutathione status (GSH) and (**b**) lipid peroxidation in treated C2C12 myotubes. Treatment groups were palmitate (PT), palmitate and tubers’ extract of orange fleshed sweet potato (PT + OSPT), palmitate and leaves’ extract of orange fleshed sweet potato (PT + OSPL), palmitate and metformin (PT + M), tubers’ extract of orange fleshed sweet potato only (OSPT), leaves’ extract of orange fleshed sweet potato only (OSPL). Different letters denote statistical significant differences between results (*p* < 0.05), Bars indicate standard deviation (*n* = 3).

In skeletal muscle, there are evidences of insulin resistance being attributable to free fatty acids [38]. Palmitate is a saturated fatty acid that has been used in different in vitro studies to create insulin resistant models [39,40]. Disruption of insulin signaling by palmitate is associated with increased intracellular oxidative stress [41]. Park et al. [39] demonstrated that treatment of C2C12 cells with palmitate increased reactive oxygen species (ROS) production and reduced insulin sensitivity as compared with untreated cells. In this study, insulin resistance in differentiated C2C12 cells was induced by incubating the cells in 0.75 mM palmitate followed by treatment with the safest maximum dosages of extracts (500 µg/mL and 100 µg/mL of OSPT and OSPL respectively) as determined by the MTT assay (Table 2). There are evidences which have demonstrated that Metformin’s ability to improve insulin sensitivity is associated with reduced mitochondrial ROS endogenous production [42]. Therefore, 1 µM metformin was used as the standard treatment in the present study. The first index of oxidative stress measured in this study was the level of reduced glutathione within the cells. Glutathione is an endogenously synthesized antioxidant and the depletion of the reduced form (GSH) is one of the indices of oxidative stress in living cells [43]. In a state of oxidative stress, GSH is utilized and converted to the oxidized form (GSSG) in order to cope with the increased ROS production. Hence, the ratio of GSH to GSSG is also an important biomarker of oxidative stress [44], although this was not measured in this study]. The GSH status of the cells was lower in the palmitate-treated group (PT) and significantly improved upon treatment with the tuber extract (PT + OSPT), leave extract (PT + OSPL) and metformin (PT + M) (Figure 2a). A decrease in the level of GSH has been associated with insulin resistance and type 2 diabetes [45]. This reduction has been attributed to an increase in oxidative stress and consequently an increased depletion of intracellular GSH in the process of scavenging free radicals and maintaining redox status within the cell [46]. In similar studies, *tert*-butyl hydroperoxide (tBHP) induced oxidative stress reduced the level of GSH in C2C12 cells which was improved significantly upon treatment with polyphenol rich-grape stem extract [47,48].

Lipid peroxidation is also an important biomarker of oxidative stress. It is the oxidative degradation of lipids caused by reactive oxygen species resulting in cellular damage [49]. Malonaldehyde (MDA) is a product of lipid peroxidation which is a useful biomarker of oxidative stress in cells [50]. Plant extracts rich in polyphenols have been linked with reduction of lipid peroxidation in different studies [51,52]. Similarly, in this study, while there was a significantly higher level of MDA in the PT group than all the other groups (Figure 2b), a significant reduction of MDA was observed upon treatments with the extracts and metformin.

#### 2.2.2. Effect of Sweet Potato Extracts on Antioxidant Enzymes

**Figure 3:**
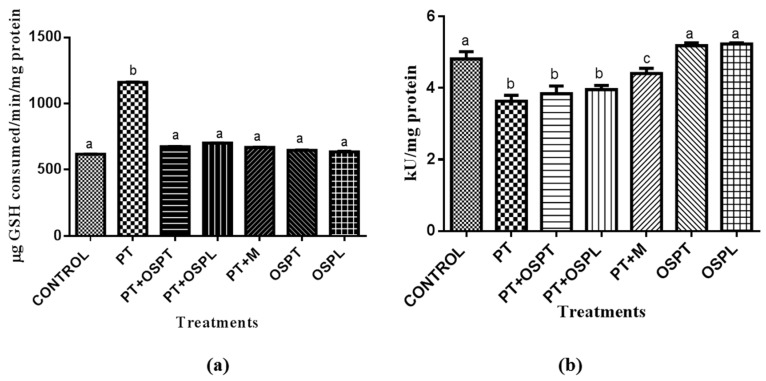
The effect of orange fleshed sweet potato extracts on (**a**) glutathione peroxidase (GPx) activity and (**b**) Catalase (CAT) activity in treated C2C12 myotubes. Treatment groups were palmitate (PT), palmitate and tubers’ extract of orange fleshed sweet potato (PT + OSPT), palmitate and leaves’ extract of orange fleshed sweet potato (PT + OSPL), palmitate and metformin (PT + M), tubers’ extract of orange fleshed sweet potato only (OSPT), leaves’ extract of orange fleshed sweet potato only (OSPL). Different letters denote statistical significant differences between results (*p* < 0.05). Bars indicate standard deviation (*n* = 3).

The activity of antioxidant enzymes are also considered indicative of the antioxidant status of a biological system [53]. Glutathione peroxidase (GPx) is an antioxidant enzyme which catalyzes the reduction of hydrogen peroxide and lipid peroxides into water and lipid alcohols respectively using glutathione as reductant [54]. In this study, as shown in Figure 3a, GPx activity was significantly higher in the palmitate-treated groups compared to all other groups. However, the activity of the enzyme reduced significantly in the extracts and metformin treated cells. A possible reason for increased GPx activity during oxidative stress is the increased ROS production, which means GPx would work more to scavenge ROS while using and depleting the intracellular GSH level [55]. On the contrary, Figure 3b shows there was a significant decrease in catalase activity in the palmitate-treated group when compared to the control group. The activity increased, although not significantly, following treatments with OSPT, OSPL and metformin. Catalase (CAT) is another important antioxidant enzyme which protects cells from oxidative damage. It has one of the highest turnover rates of all enzymes and catalyzes the decomposition of hydrogen peroxide into water and oxygen [56].

#### 2.2.3. Effect of Sweet Potato Extracts on Antioxidant Capacity

FRAP and TEAC assays are common measure of antioxidant capacity relative to a standard antioxidant compound. FRAP and TEAC values of the palmitate treated groups were significantly lower and there were significant improvements of both values following treatment with the extracts and metformin (Table 3). The increased antioxidant capacity was expected due to the improved total glutathione status and antioxidant enzyme activity as well as reduced lipid peroxidation observed in the treated cells.

### 2.3. Effect of Sweet Potato Extracts on Expression Levels of Glut4, Nrf1, Mef2a, Cpt1 and Acc2 Genes

**Figure 4:**
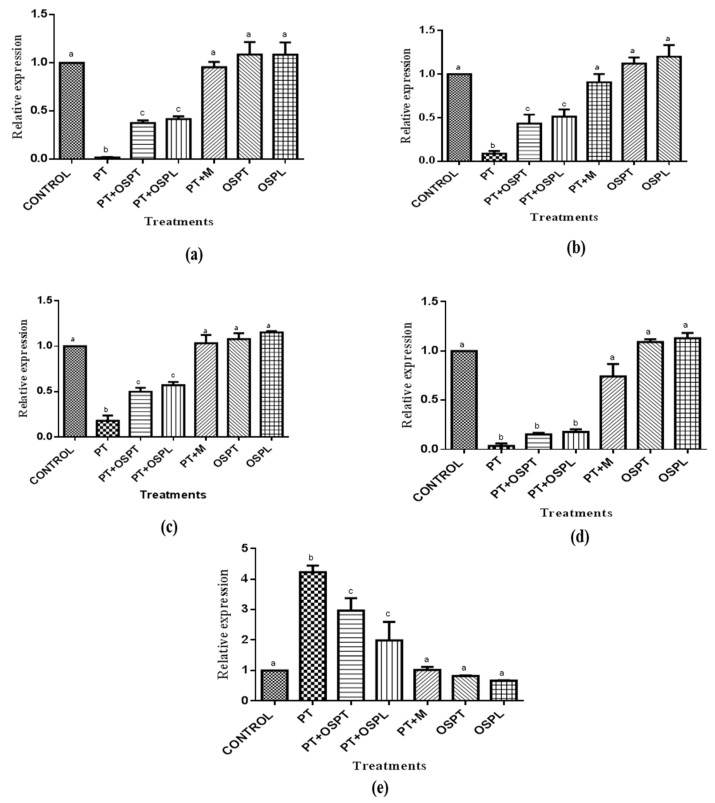
The effect of orange fleshed sweet potato extracts on the expression of (**a**) Glucose transporter 4 (*glut4*) (**b**) Nuclear respiratory factor 1 (*nrf1*) (**c**) Mycocyte enhance factor 2A (*mef2a*) (**d**) Carnitine palmitoyl transferase 1 (*cpt1*) and (**e**) Acetyl CoA carboxylase 2 (*acc2*) in treated C2C12 myotubes. Treatment groups were palmitate (PT), palmitate and tubers’ extract of orange fleshed sweet potato (PT + OSPT), palmitate and leaves’ extract of orange fleshed sweet potato (PT + OSPL), palmitate and metformin (PT + M), tubers’ extract of orange fleshed sweet potato only (OSPT), leaves’ extract of orange fleshed sweet potato only (OSPL). Results are expressed as relative expression of mRNA with respect to untreated controls. Different letters denote statistical significant differences between results (*p* < 0.05). Bars indicate standard deviation (*n* = 3).

The expression levels of the genes (*glut4*, *nrf1*, *mef2a*, *cpt1* and *acc2*) were determined relative to a housekeeping gene (*gapdh*) and as a fold change in expression of the control group. The results (Figure 4) showed that there were significant decreases in expression of *glut4*, *nrf1* and *mef2a* in the palmitate treated group when compared with the control group. However, there were significant improvement in the genes’ expression in the PT + OSPT and PT + OSPL groups when compared to the PT group, although the expressions were still significantly lower when compared to the untreated CONTROL and PT + M groups. The relative expressions of these genes were improved near normal in the PT + M group as they were not significantly different from the untreated CONTROL group. *Glut4* is an insulin-sensitive major transporter of glucose in the skeletal muscle. It is a key glucose transporter which plays an important role in the maintenance of glucose homeostasis and hence, an important pharmacological target in the management of type 2 diabetes [8]. Insulin resistance is associated with a reduced expression of *glut4* gene and its protein [57,58]. *Mef2a* and *nrf1* are transcription factors that both play important roles in the regulation of *glut4* expression and ultimately glucose uptake metabolism [9,59]. The expression and activity of *glut4* gene have been found to be directly correlated with that of *nrf1* and *mef2a* as well as with insulin sensitivity and the maintenance of glucose homeostasis in skeletal muscle. Therefore, the improved expressions of these genes in the treated cells indicate the potential of aqueous-methanol extracts of orange fleshed sweet potato to improve insulin sensitivity.

Furthermore, Figure 4c,d show there were significant decrease in expression of *cpt1* and increase in expression of *acc2* respectively in the palmitate treated C2C12 cells when compared with the untreated control group. However, an increase in the expression of *cpt1*, although not significantly, and a significant decrease in the expression of *acc2* up to 3 and 2 folds were observed in the PT + OSPT and PT + OSPL groups respectively. The relative expression of *cpt1* and *acc2* in the PT + M group was not significantly different from the untreated CONTROL group. *Acc2* and *cpt1* are important regulators of mitochondrial fatty acid oxidation and hence strategies that influence their expression would affect the level of intracellular lipids and have therapeutic implications in the management of insulin resistance [60]. Accumulation of intracellular lipids has been implicated in the development of insulin resistance in skeletal muscle [37]. Choi and colleagues [61] reported increased fatty acid oxidation, reduced diacylglycerol content and improved insulin sensitivity in *acc2* knockout mice. This is consistent with earlier findings of Rosa et al. [62] which attributes the reversal of insulin sensitivity to the downregulation of *acc2* gene in the skeletal muscle of previously obese subjects after undergoing bariatric operation. Also, overexpression of *cpt1* in skeletal muscle resulted into improved insulin sensitivity in high-fat diet induced insulin resistance [14]. Similarly, in this study, the increased and decreased expressions of *cpt1* and *acc2* respectively observed in the treated groups further suggest that aqueous-methanol extracts of orange fleshed sweet potato have the potential to improve insulin sensitivity in insulin resistant skeletal muscle. In the OSPT and OSPL groups, there was higher expression of all the genes although not significant (*p* < 0.05) when compared to that of untreated control.

It is noteworthy that treatments with the extracts at the safest maximal dosages did not positively modulate the genes and alleviate oxidative stress as much as treatments with Metformin, a standard antidiabetic drug. This is probably because the extracts were in their crude forms and hence, the presence of inactive compounds lowers the concentration of the bioactive ones and the potency of the extracts. Isolation of the active contents of the extracts would help to further explore the antidiabetic potential of sweet potato. Nonetheless, the findings of this study provide insight into the potential of the extracts of orange fleshed sweet potato as useful resources in the development of phytotherapy agent against insulin resistance and type 2 diabetes.

## 3. Materials and Methods

### 3.1. C2C12 Cells Subculture and Differentiation

C2C12 cells, a kind gift from Prof E.O Ojuka’s laboratory (Division of Exercise Science and Sport Medicine, University of Cape Town, Cape Town, South Africa) were stored and maintained in freezing media containing 70% Dulbecco’s Modified Eagle Medium (DMEM), 20% fetal bovine serum (FBS) and 10% dimethyl sulfoxide (DMSO) in −80 °C and liquid nitrogen. In all the assays except the cytotoxicity assay, C2C12 cells at a density of 2.2 × 10^6^ were plated in 100 mm petri dishes containing 10 mL of DMEM supplemented with 10% FBS and 1% antibiotics and incubated at 37 °C in a 5% CO_2_ atmosphere. At about 70% confluency, C2C12 cells were differentiated for 96 h by adding differentiation media containing 97% DMEM, 2% FBS and 1% antibiotics. The media was changed every 24 h until formation of myotubes were observed.

### 3.2. Plant Samples and Preparation of Extracts

The cuttings of the orange fleshed sweet potato cultivar ‘Bophelo’ (accession number-2002-21-1) were obtained from Agricultural Research Council (ARC, Roodeplaat, South Africa) and cultivated at the North West University research farm (Mafikeng campus, Mmabatho, South Africa). Freshly harvested leaves and tubers were rinsed under running water; the leaves were cut into pieces and tubers into thin chips before being air dried in the laboratory. Air dried samples were grounded into fine powder and packed in an air tight container. Preparation of crude aqueous and aqueous-methanol (1:1) extracts of the tubers and leaves were done by soaking the powder in the respective solvents (5 g/100 mL) for 24 h at room temperature with constant shaking. Extracts were recovered through lyophilization and evaporation using Alpha 1–4 LSC Plus freeze dryer (Martin Christ, Gefriertrocknungsanlagen GmbH, Osterode am Harz, Germany) and RE-52A rotary evaporator (Shanghai YARONG Biochemistry Instrument Factory, Shanghai, China) respectively.

### 3.3. Quantification of Specific Polyphenols by HPLC Analysis

Specific flavonoids (catechin, hyperoside, isoorientin, kaempferol, orientin, rutin, quercetin and vitexin) and phenolic acids (caffeic acid, protocatechuic acid, isovanillic acid and vanillic acid) were quantified in the extracts using HPLC. Stock solutions (100 µg/mL) of the standards were prepared by dissolution in distilled water except for kaempeferol which was dissolved in a mixture of water, isopropanol and acetonitrile. Dried extracts were reconstituted to make 1 mg/mL and 10 mg/mL extracts of leaves and tubers respectively in the mobile phase solvent and filtered through a 0.45 µm syringe filter system before injection into the LC system.

HPLC analysis was conducted on Agilent 1200 LC system (Agilent Technologies, Santa Clara, CA, USA). Separation was achieved by injecting one microliter of sample into a Waters HSS T3 column (2.1 × 100 mm, 1.8 µm) (Microsep, Bramley, Johannesburg, South Africa), at 30 °C. The mobile phases were comprised of water (solvent A) and acetonitrile (solvent B), each containing 0.1% formic acid. Mass spectrometry detection is performed on an Agilent 6410 Triple Quadrupole instrument using positive electrospray ionisation. The drying gas temperature is used at 300 °C with a drying gas flow of 7.5 L/min and nebuliser pressure of 30 psi. Mass spectrometry conditions were optimised with the MassHunter optimizer software (B.04.01) (Agilent Technologies, Santa Clara, CA, USA) using the standards. Detected peaks were identified and quantified by comparing the retention time and peak area to that of known standards.

### 3.4. MTT Cytotoxicity Assay

C2C12 cells were plated at a density of 5 × 10^4^ in a 96 well plate. After 48 h differentiation, cells were treated with different concentrations (10, 20, 50, 100, 200, 500, 1000 µg/mL) of OSPT and OSPL in growth medium for 24 h at 37 °C. MTT assay was carried out using the Vybrant^®^ MTT Cell proliferation assay kit (Molecular Probes, Carlsbad, CA, USA) according to the manual. Cell viability was expressed as percentage and estimated as:

(1) Absorbance of sample−absorbance of sample blank/Absorbance of control−Absorbance of sample blank×100

### 3.5. Preparation of Palmitate and Induction of Insulin Resistance

Palmitate was prepared in 50% ethanol solution heated to 95 °C [31]. Prior to treatment with palmitate, cells were serum and glucose starved by incubating in PBS for 30 min at 37 °C and in 5% CO_2_. Insulin resistance was induced by incubating the cells with DMEM containing 0.75 mM palmitate [40,63,64] and 2% BSA for 16 h.

### 3.6. Treatment of C2C12 Cells with Sweet Potato Extracts

Differentiated cells were divided into seven groups and treated according to Table 4. After 16 h of incubation with palmitate, the media was changed and the cells were washed thrice in PBS. The cells were then exposed to 500 µg/mL and 100 µg/mL of OSPT and OSPL respectively in base DMEM supplemented with 2% BSA for 3 h. Untreated cells served as negative control while cells treated with 1 µM metformin (40) for 3 h served as positive control. Treatment with only OSPT and OSPL were included to assess the protective effect of the extracts on healthy C2C12 skeletal muscle cells.

### 3.7. Oxidative Stress and Antioxidant Activity Assays

#### 3.7.1. Bradford Protein Assay

Prior to each assay, protein determination was carried out on the cell homogenates to normalize the assessment of the level of oxidative stress and antioxidant activity across the treated cells. Cells were seeded into 6-well plates at a density of 0.3 × 10^6^. Differentiated cells were treated as described in Section 3.1 and harvested by scraping with sterile PBS into Eppendorf tubes. The cells were then homogenized on ice for about 2 min. The homogenates were centrifuged at 4 °C for 15 min. The supernatants were removed and kept on ice for use in the assay. Five microliters (5 µL) of the supernatants or protein standards (0.2–1.0 mg/mL) followed by 250 µL of Bradford reagent was added in triplicates to the wells of 96 well plate. The absorbances of the samples were read at 595 nm after incubation for 5 min. The net absorbance of the standards was plotted against the concentrations to generate a standard curve. The protein concentrations of the samples were extrapolated from the standard curve.

#### 3.7.2. Glutathione Status and Glutathione Peroxidase Activity Assays

These were done according to the method of Rotruck et al. [65] using reduced glutathione as standard. 100 microlitres (100 µL) of 0.3 M K_2_HPO_4_ and 50 µL of 0.04% DTNB was added to 50 µL of the cell homogenate or EDTA solutions standards (25, 50, 100, 150, 200 µM). Absorbance of the reaction mixture was read at 412 nm against a blank and the concentrations of the standards were plotted against the absorbances. Reduced GSH levels of the samples were estimated from the generated standard curve. To estimate the GPx activity, a reaction mixture (500 µL potassium phosphate buffer (pH 7.5), 100 µL 10 mM NaN_3_, 200 µL 4 mM GSH, 100 µL 2.5 mM H_2_O_2_, 500 µL H_2_O, 600 µL sample) was prepared. The mixture was incubated at 37 °C for 3 min followed by the addition of 0.5 mL of 10% TCA and centrifugation at 3000 rpm for 5 min. 100 µL of 0.3 M K_2_HPO_4_ and 50 µL of 0.04% DTNB was added to 50 µL of the supernatant or standards. Absorbance of the reaction mixture was read at 412 nm against a blank. The concentration of the remaining GSH was extrapolated from the standard curve. GPx activity was expressed as µg of GSH consumed/mg of protein and calculated by the formulas;

(2) GSH consumed=245.34−GSH remaining

(3) Glutathione peroxidase activity=Amount of GSH consumed/mg protein

#### 3.7.3. Catalase Activity Assay

CAT activity was measured by the method of Hadwan [66]. One hundred microliters (100 µL) of sample was added to 1000 µL of H_2_O_2_, the mixture was vortexed and incubated for 3 min after which dichromate/acetic acid (2 mL) was added to each test. The control and standard tests had distilled water in place of H_2_O_2_ and sample, respectively. Thereafter, the tubes were kept at 100 °C for 10 min and centrifuged at 2500 g for 5 min after cooling to remove precipitated protein. Changes in absorbances were recorded at 570 nm against the blank sample. CAT activity was calculated by the equation:

(4) 2.303/t×{log S°/S−M}×Vt/Vs

where: t: time taken for the reaction, S^°^: Absorbance of standard tube, S: Absorbance of test tube, M: Absorbance of control test, Vt: Total volume of reagents in test tube and Vs: Volume of serum

#### 3.7.4. Lipid Peroxidation Assay

The extent of lipid peroxidation across the treatment groups were measured using the thiobarbituric acid reactive substances (TBARS) assay [67]. One hundred microliters (100 µL) of cell homogenates or standards (2, 4, 6, 8, 10 µM of MDA solution) were added to 1 mL of 0.67% (*w*/*v*) TBA, 1 mL of 20% (*w*/*v*) trichloroacetic acid (TCA) and 1.5 mL of 0.04% butylated hydroxytoluene (BHT) in microtubes. The mixture was incubated in boiling water for 20 min and then cooled to room temperature. Thereafter, the tubes were centrifuged at 4000 g for 10 min, the supernatants were collected and the changes in absorbances were measured at 532 nm against blank. The concentrations of MDA were extrapolated from the standard curve obtained by plotting the absorbances of the standards against their respective concentrations and using the formula;

(5) Amount of MDA of sample/mg of protein

Results were expressed as µmol MDA/mg protein

#### 3.7.5. Antioxidant Activity Assays

Antioxidant activity was determined by using FRAP [68] and TEAC [69] assays. FRAP reagent was prepared by mixing 30 mL acetate buffer, 3 mL TPTZ solution, 3 mL FeCl_3_ solution and 6.6 mL distilled water. Aqueous solutions of 0, 25, 50, 100 and 200 µM ascorbic acid were used as standards. 10 µL of the cell homogenate/standards and 300 µL of FRAP reagent was added to the well and incubated at 37 °C for 30 min. Absorbances were read at 593 nm. Results are expressed as µmol AAE/mg protein. TEAC assay was done by reacting 88 µL of the potassium-peroxodisulphate solution (7 mM) and 5 mL of the ABTS solution (140 mM) to produce ABTS radical cations. The mixture was left in the dark at room temperature for 24 h and then diluted with ethanol in ratio 1:20 before use. 25 µL of the sample/standards was added to 300 µL of diluted ABTS and incubated for 30 min at room temperature. The absorbances were read at 734 nm. A standard curve was prepared by using 0, 25, 50, 100, 200, 500 µM solution of Trolox in ethanol.

### 3.8. Total RNA Extraction and cDNA Synthesis

Total RNA extraction from the cells and purification was carried out using PureLink^®^ RNA mini kit and according to the manufacturer’s manual. Integrity of extracted RNA was confirmed by 1% agarose gel electrophoresis. The extracted mRNA was reverse-transcribed into first-strand cDNA using a Superscript^TM^ VILO^TM^ Mastermix. The synthesized cDNA was stored in −20 °C for onward use in qPCR reactions.

### 3.9. Quantitative Reverse Transcription Polymerase Chain Reaction (qRT-PCR) Analysis

Real Time qRT-PCR was carried using a Steponeplus^TM^ PCR machine (Applied Biosystems, Foster City, CA, USA) and the PowerUp^TM^ SYBR^TM^ Green Master Mix (Applied Biosystems). The genes of interest studied were; (*glut4 nrf1*), *mef2a*, *cpt1* and *acc2*. GAPDH was used as reference gene. The reaction mix of the qRT-PCR reactions contains approximately 10 ng (2 µL) of cDNA from each treatment group and per each gene of interest and the reference gene. Specific primers (forward and reverse) designed for amplification of the genes (Table 5) were also included in the reaction mix. No template control (NTC) reactions (no cDNA in reaction mix) were also set up to detect PCR contaminations. Relative gene expression was analyzed by the comparative ΔCT method using the formula, 2^−ΔΔCT^.

### 3.10. Statistical Analysis

All assays were done in triplicates and results data are presented as mean ± standard deviation. Statistical analysis was done using the GraphPad prism 5 statistical package (GraphPad Software, La Jolla, CA, USA), and significant differences among the samples were calculated using one-way ANOVA followed by Tukey’s test at *p* < 0.05.

## 4. Conclusions

With the exception of a few compounds, the aqueous-methanol extracts of the leaves of the orange fleshed sweet potato mostly had higher concentrations of the specific polyphenols measured which also translated into higher bioactivity of the leaves’ extracts although not significantly. The improved antioxidant system in the treated cells which was accompanied by the positive modulation of the genes suggests that the aqueous-methanol extracts of both the leaves and tubers of orange fleshed sweet potato has antidiabetic potential which was mediated by their antioxidant capacity. Further studies which involve the assessment of the effect of the extracts on glucose uptake and GLUT 4 translocation in an insulin resistant model is important to establish the therapeutic benefit in type 2 diabetes. Pharmacokinetics and bioavailability studies in an in vivo model are also necessary in the design of safe dosage regimens of the extracts and/or its active compounds. 

## Figures and Tables

**Table 1:** Quantification of phenolic acids and flavonoids in aqueous and aqueous-methanol extracts of orange fleshed sweet potato.

Compound	Retention Time (min)	Parent Ion (*m*/*z*)	Product Ion (*m*/*z*)	Amount of Phenolic Acids and Flavonoids (mg/g of Extract)			
				LEAVES		TUBERS	
				AQ	AQ-ME	AQ	AQ-ME
Caffeic acid	14.59	181	163	0.98 ± 0.003	3.76 ± 0.003	1.7 ± 0.000	19.4 ± 0.042
Catechin	16.6	291.1	139	ND	0.01 ± 0.010	ND	ND
Hyperoside	15.69	465.1	303.1	1.39 ± 0.003	139.83 ± 0.006	0.22 ± 0.007	9.78 ± 0.003
Iso-orientin	14.8	449.1	299	ND	ND	ND	ND
Isovanillic acid	10.9	169	65.1	0.04 ± 0.005	0.13 ± 0.003	0.14 ± 0.007	0.04 ± 0.000
Kaempferol	15.8	287.1	167.2	ND	0.09 ± 0.007	ND	ND
Orientin	14.7	449.1	329	ND	ND	ND	ND
Protocatechuic acid	10.45	155	65.1	0.45 ± 0.004	1.22 ± 0.014	2.5 ± 0.008	0.08 ± 0.003
Quercetin	15.65	303.1	153	0.28 ± 0.003	23.36 ± 0.000	0.03 ± 0.001	1.76 ± 0.004
Rutin	15.34	611.2	303	0.73 ± 0.007	69.67 ± 0.007	ND	2.91 ± 0.007
Vanyllic acid	9.854	169	65.1	0.26 ± 0.0023	0.4 ± 0.014	0.45 ± 0.002	0.81 ± 0.127
Vitexin	15.4	433.1	313	ND	ND	ND	ND

Values are mean ± standard deviation (*n* = 2). AQ—aqueous extracts, AQ-ME—aqueous-methanol extracts, ND—not detected.

**Table 2:** Percentage viability of C2C12 cells treated with different concentrations of OSPT and OSPL.

Concentration (µg/mL)	OSPT	OSPL
	Cell Viability (%)	Cell Viability (%)
10	97.20 ± 1.56 ^a^	96.14 ± 0.08 ^a^
20	96.12 ± 1.04 ^a^	95.12 ± 1.72 ^a^
50	91.47 ± 2.16 ^a^	93.88 ± 0.09 ^a^
100	90.28 ± 2.12 ^a^	90.96 ± 2.34 ^a^
200	90.09 ± 1.87 ^a^	78.23 ± 1.11 ^b^
500	91.37 ± 1.68 ^a^	75.16 ± 1.46 ^b^
1000	76.31 ± 2.01 ^b^	77.91 ± 0.65 ^b^

Values are mean ± standard deviation (*n* = 3). Bars with different letters denote statistical significant differences within the same column (*p* < 0.05). OSPT, Aqueous-methanol extract of tubers of orange fleshed sweet potato; OSPL, aqueous-methanol extract of leaves of orange fleshed sweet potato.

**Table 3:** The effect orange fleshed sweet potato extracts on antioxidant capacity (FRAP and TEAC Values).

Treatment Groups	FRAP Values (µM AAE/mg Protein)	TEAC Values (µM TE/mg Protein)
CONTROL	271.0 ± 4.17 ^a^	107.2 ± 1.68 ^a^
PT	102.2 ± 5.06 ^b^	39.8 ± 1.80 ^b^
PT + OSPT	167.7 ± 0.54 ^c^	71.94 ± 8.0 ^c^
PT + OSPL	172.1 ± 1.94 ^c^	76.19 ± 7.6 ^c^
PT + M	251.3 ± 2.50 ^a^	91.06 ± 1.40 ^a^
OSPT	299.8 ± 2.5 ^d^	127.9 ± 2.10 ^d^
OSPL	296.9 ± 7.4 ^d^	126.3 ± 2.51 ^d^

Different letters down a column denote statistical significant differences between results (*p* < 0.05), (*n* = 3). PT, palmitate; OSPT, tubers’ extract of orange fleshed sweet potato; OSPL, leaves’ extract of orange fleshed sweet potato; M, metformin.

**Table 4:** Treatment groups for C2C12 cells.

Treatment Groups	Palmitate (PT)	Tuber Extracts (OSPT)	Leaf Extracts (OSPL)	Metformin (M)
CONTROL	−	−	−	−
PT	+	−	−	−
PT + OSPT	+	+	−	−
PT + OSPL	+	−	+	−
OSPT	−	+	−	−
OSPL	−	−	+	−
PT + M	+	−	−	+

+, Treated with; −, not treated with.

**Table 5:** Primers sequences for quantitative reverse transcription polymerase chain (qRT-PCR) reaction.

Gene	Primer Sequence(5′-3′)
*glut4*	Forward-AAGATGGCCACGGAGAGA Reverse-GTGGGTTGTGGCAGTGAGTC
*nrf1*	Forward-AAACACAAACTCAGGCCACC Reverse-CCATCAGCCACAGCAGAGTA
*mef2a*	Forward-GTGTACTCAGCAATGCCGAC Reverse-AACCCTGAGATAACTGCCCTC
*cpt1*	Forward-CCAGGCTACAGTGGGACATT Reverse-GAACTTGCCCATGTCCTTGT
*acc2*	Forward-GTCCTCATCATGAACGGCTG Reverse-AGGACAGTGGGGTCGTTTTC
*gapdh*	Forward-GCACAGTCAAGGCCGAGAAT Reverse-GCCTTCTCCATGGTGGTGAA
